# Evaluation of using grip strength and hand muscle cross-sectional area to predict secondary fractures post distal radius fracture

**DOI:** 10.1007/s11657-024-01465-5

**Published:** 2025-01-16

**Authors:** Matthew Tsz Kin Kong, Christian Fang, Colin Shing Yat Yung, Theresa Kwok, Keith Leung, Frankie Leung

**Affiliations:** 1 Li Ka Shing Faculty of Medicine, The University of Hong Kong, 21 Sassoon Rd, Pok Fu Lam, Hong Kong, China; 2 Department of Orthopaedics and Traumatology, Queen Mary Hospital The University of Hong Kong, 102 Pok Fu Lam Road, Hong Kong, China; 3 Occupational Therapy Department, David Trench Rehabilitation Centre, 1F High Street, Hong Kong, China

**Keywords:** Sarcopenia, Osteoporosis, Osteosarcopenia, Wrist fracture, Distal radius fracture, Colles fracture, Secondary fracture, Recurrent fracture, Grip strength

## Abstract

**Summary:**

Grip strength measurement, as a surrogate of sarcopenia diagnosis, effectively predicts secondary fracture risk in distal radius fracture patients. This simple tool enhances clinical practice by identifying high-risk patients for targeted interventions, potentially preventing or reversing functional decline and recurrent fractures.

**Purpose:**

To evaluate grip strength and hand muscle cross-sectional area as predictors of secondary fracture risk in patients with a history of distal radius fracture (DRF), serving as surrogates of the diagnosis of sarcopenia.

**Methods:**

A retrospective cohort study of 745 DRF patients was analyzed with their grip strength data using Cox proportional hazards regression, receiver operating characteristic analysis, and Kaplan–Meier analysis to predict secondary fracture risk over an average of 12 years. Hand muscle cross-sectional area was similarly analyzed.

**Results:**

Patients with a history of DRF were predicted to have a 4.1% higher likelihood of experiencing a secondary fracture per kilogram reduction in their grip strength (*p* < 0.008), independent of age and sex. Patients were categorized as high-risk (≤ 16 kg), moderate-risk (17–24 kg), or low–risk (≥ 25 kg) (*p* < 0.001). High-risk patients showed a 2.2-fold (95% CI = 1.55–3.17) higher recurrent fracture risk compared to low-risk patients. Cumulative secondary fracture probabilities of the high-risk group patients at 5, 10, and 15 years were estimated to be 16%, 30%, and 54%, respectively.

**Conclusions:**

Grip strength measurement, as a surrogate of sarcopenia diagnosis, effectively predicts secondary fracture risk in patients with DRF. This simple tool could improve clinical practice by identifying high-risk patients for targeted interventions to prevent recurrent fractures or even reverse functional decline.

## Introduction

Distal radius fractures (DRFs), commonly known as Colles fractures or wrist fractures, are the most prevalent type of upper limb fractures [1–3], especially among osteoporotic patients [2, 4]. DRFs resulting from low-energy trauma, such as ground-level falls [3, 5], are classified as fragility fractures and are typically associated with osteoporosis [6]. While DRFs are not usually associated with significant morbidity or mortality [4, 7], they serve as a critical indicator for the risk of secondary fractures, especially in older adults with osteoporosis [1, 7]. A 2022 retrospective study of 705 patients found that 7.9% experienced subsequent fractures within an average follow-up period of 33.5 months post-DRF [8]. Research showed that DRFs present a crucial opportunity for healthcare providers to implement early interventions to slow fragility deterioration and prevent potential fractures in the near future [1, 3, 7]. However, despite the well-established link between osteoporosis and recurrent fractures, many doctors still lack awareness about administering osteoporotic treatments to their patients [9].

Sarcopenia, like osteoporosis, is a significant age-related condition linked to a higher risk of adverse clinical outcomes [10–12], including falls [10, 12, 13] and fractures [7, 10, 12]. Notably, sarcopenia and osteoporosis frequently coexist, a combination termed “osteosarcopenia” [14]. Their coexistence can have a synergistic effect on the associated complications [15, 16] due to their overlapping pathophysiology [17, 18]. While osteoporosis has a clear diagnostic threshold through dual X-ray absorptiometry (DEXA) [19], sarcopenia’s definition and diagnostic criteria remain variable and inconsistent [20–22]. Nonetheless, diagnosing sarcopenia, in general, involves evaluating muscle mass, muscle strength, and physical performance [23–25]. Unfortunately, these measurements are rarely performed in clinical practice [11, 26, 27], particularly in this locality.

Grip strength is a widely used measure to assess muscle strength in sarcopenia diagnosis [23–25]. It is a non-invasive, simple, quick, and cost-effective way to evaluate muscle strength [26, 28] that also represents lower extremity muscle power [29]. Studies have linked low grip strength to various adverse outcomes [25, 26, 28], including recurrent falls [3, 13, 20] and fragility fractures [30–32], independent of other sarcopenia diagnostic parameters. These findings have become our interest in investigating whether grip strength measurement alone, as a surrogate for sarcopenia diagnosis, can predict the risk of re-fracture in patients with previous DRF.

Bones and muscles should be regarded as a unified system because their functions and structures are intricately connected [33, 34]. This perspective emphasizes the importance of addressing osteoporosis and sarcopenia simultaneously through effective osteoporosis management [10, 35]. Research has shown that sarcopenia can be reversed [12, 36], highlighting the significance of early detection to enable timely interventions that can prevent recurrent fractures or even reverse functional decline.

While DEXA is used to diagnose both osteoporosis and sarcopenia in the form of muscle mass [23–25], its use is limited by the cost and availability in clinical settings [25, 27]. Conversely, patients with wrist fractures, particularly those undergoing surgical interventions, typically have their computed tomography (CT) scans accessible in the medical databases. Therefore, we suggested that the cross-sectional areas of hand muscles, including the thenar and hypothenar muscles, can be visualized and quantified in transverse CT images. This serves as a proxy for muscle mass to aid in sarcopenia diagnosis and to predict the risk of subsequent fractures.

**Fig. 1:**
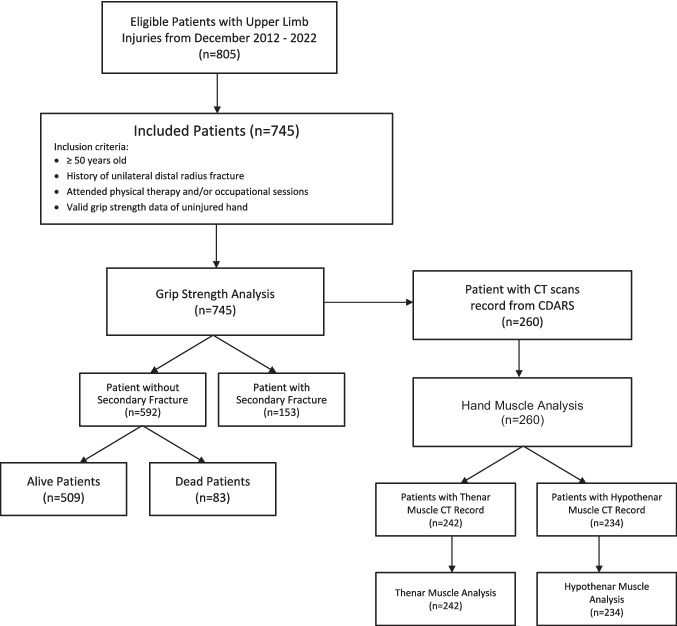
Design of the research study

Given the limitation of DEXA scans in clinical settings, there is a growing need for a straightforward and rapid screening tool to identify patients with distal radius fractures (DRFs) who are at risk of secondary fractures and need optimal stratified care. Considering the bidirectional relationship between osteoporosis and sarcopenia, screening for one condition likely indicates the presence of the other. Therefore, we hypothesize that measuring grip strength and the cross-sectional area of hand muscles could effectively predict the risk of secondary fractures in patients with a history of DRF, without relying solely on sarcopenia diagnosis (Fig. 1).

## Method

Patients admitted to a university-affiliated tertiary hospital from December 2012 to 2022 were retrospectively reviewed. Data were retrieved from the rehabilitation center database according to the following criteria. Inclusion criteria were patients aged ≥ 50 years old with a history of unilateral DRF, attendance at the physical therapy and/or occupational therapy sessions, and grip strength measured on an uninjured hand by a dynamometer (JAMAR Hydraulic Hand Dynamometer 5030J1, Sammons Preston Inc, IL, USA). Patients were excluded if they were cognitively incompetent or had a pathological fracture.

The cross-sectional area (mm^2^) of patients’ thenar and hypothenar muscles were measured on their computed tomography (CT) scans. These scans were generally available from a subgroup of DRF patients undergoing surgical treatments and were accessed electronically via the region-wide picture archiving and communication system (PACS) database. The cross-sectional area of the thenar and hypothenar muscles was measured on transverse CT scans at the level of the midcarpal joint (between the proximal and distal role of carpal bones). Due to the variations in hand positioning, thenar and hypothenar muscles not perfectly visualized at the midcarpal joint level were excluded. A trained observer measured the cross-sectional areas twice, and intra-observer reliability was validated by the intraclass correlation coefficient.

**Fig. 2:**
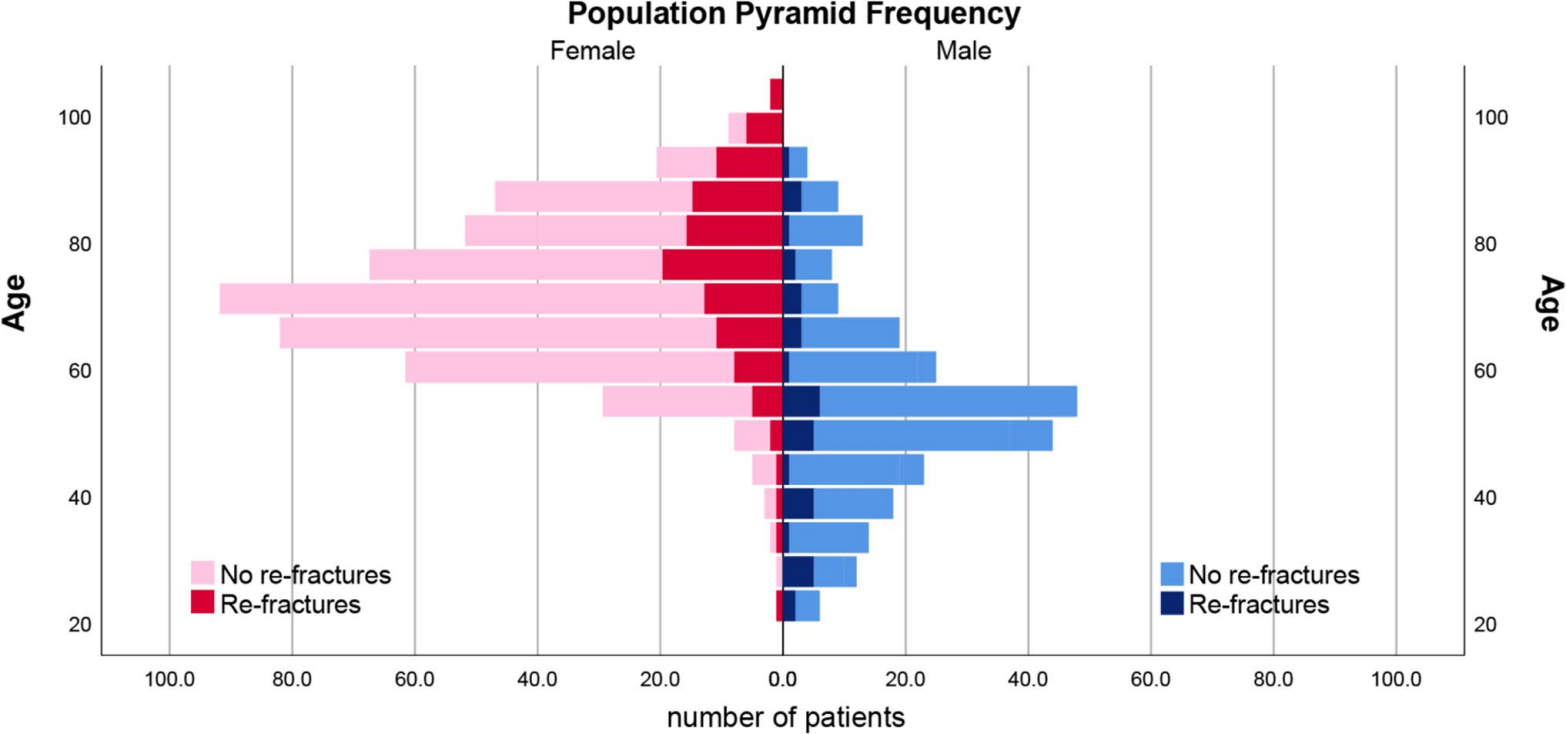
Demographic representation of all patients with and without re-fractures

Clinical Data Analysis and Reporting System (CDARS) [37], which is a region-wide database for all public hospitals, was electronically queried to identify secondary fractures following the initial DRF. According to a previous study on CDARS, the querying system used in our study research estimated an overall coverage positive predictive value (PPV) of 96.8%, with a 100% PPV for hip, humerus, forearm, and wrist fracture. This demonstrated CDARS as a valid system with high PPV for identifying and screening osteoporotic fractures, including wrist fractures [37]. Secondary fracture events were identified from subsequent clinical encounters and coded using the International Classification of Diseases 9th revision system (ICD-9: 733 & 800–829, pertaining to the keyword “fractures”). All types of fractures included in our analysis were summarized in Appendix Table 5. A secondary fracture was defined as any subsequent fracture after the initial DRF, regardless of the type or the duration post-DRF. The main outcome measure for secondary fracture was binary: either the presence or absence of a secondary fracture for each patient. The patients’ latest clinical follow-up dates were retrieved from CDARS to indicate survival and electronic medical record updates. Mortality data was electronically compiled from the region-wide death registry. The study’s endpoint was the occurrence of any secondary fracture, and patient loss to follow-up, including death, was considered as an alternative endpoint, with data censored in the Kaplan–Meier analysis. Only the first instance of a re-fracture can be counted per patient, and subsequent re-fractures (tertiary or quaternary) were not included in this study. All the statistical analyses were performed by the SPSS statistical software (Version. 26, IBM, Armonk, USA) (Fig. 2).

## Statistical analysis

**Fig. 3:**
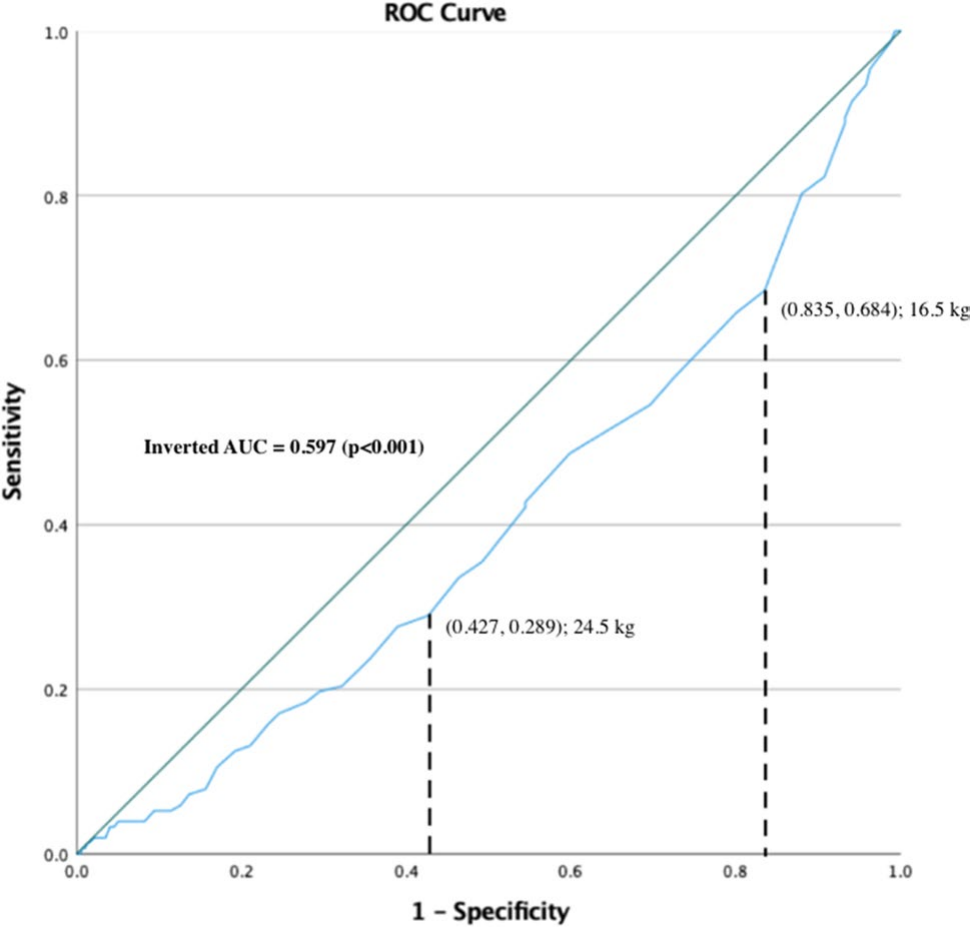
Receiver operating characteristic (ROC) analysis on grip strength data

This study utilized a comprehensive statistical approach to evaluate predictors of secondary fractures. Receiver operating characteristic (ROC) analysis was performed to stratify patients based on grip strength and hand muscle cross-sectional areas (the thenar and hypothenar muscles), as the two main predictors of secondary fractures (Fig. 3, Appendix Fig. 8). Thresholds identified from ROC analysis were used to calculate mean durations and relative risks (RRs) for secondary fractures, and their significance was calculated. An independent *t*-test was performed to examine the significance of these predictors and their ability to predict secondary fractures. The correlation between grip strength and muscle cross-sectional area was evaluated using scatter plots and the Pearson coefficient. Kaplan–Meier analysis was used for the time-to-event (TTE) analysis of secondary fractures, with curves compared using the log-rank (Mantle-Cox) method. The same procedures were performed on sex and age as univariate to determine their ability as an independent risk predictor for re-fracture (Appendix Figs. 6 and 7). A multivariate Cox proportional hazards regression analysis was performed to identify the most significant and independent predictor of secondary fractures, considering age, sex, and grip strength as covariates. All analyses used a significance level of *p*-value < 0.05.

## Results

The study included 745 patients (66.2% females) with an average age of 70.2 years old. Among the 745 patients, 153 patients (20.5%; 15.17% females) had experienced at least one episode of secondary fracture (Table 1). The average duration of secondary fracture occurrence was 70.8 months post-DRF (range = 0–217 months, SD = 59.4). The remaining 592 patients (79.5%; 51.01% females) had no record of any secondary fractures. The mean uninjured grip strength and thenar and hypothenar muscle cross-sectional areas are listed in Table 1. The demographic distribution of all patients by age and sex and the presence of re-fractures are illustrated in Fig. 2.

**Table 1:** Demographics and descriptive data of the research cohort

	*n*	*Age*	*Grip Strength*	*Thenar Area*	*Hypothenar Area*
**Patients with distal radius fracture (>*n***= 745)					
Male	252 (33.8%)	63.3	34.1	207.4	137.7
Female	493 (66.2%)	72.9	19.6	128.0	81.0
**Patients with Re-Fracture**(***n*** = 153; 20.5%)					
Male	40 (5.37%)	64.2	32.1	146.7	105.6
Female	113 (15.17%)	77.8	18.5	84.9	51.0
**Patients without Re-fracture** (***n*** = 592; 79.5%)					
Male	212 (28.46%)	63.4	34.5	219.4	144.3
Female	380 (51.01%)	72.4	19.9	140.5	89.8
**Patients without Re-fracture, Alive **(***n*** = 509; 68.3%)					
Male	187 (25.1%)	61.7	35.5	234.0	155.2
Female	322 (43.2%)	71.2	20.3	156.5	99.9
**Patients without Re-fracture, Dead** (***n*** = 83; 11.1%)					
Male	25 (3.4%)	75.7	27.1	107.0	60.4
Female	58 (7.8%)	79.0	17.7	54.3	37.1

Patients with lower grip strength, older age, and female sex showed a higher risk of secondary fractures. Analysis of these factors revealed significant associations with secondary fracture risk. The ROC analysis (inverted AUC = 0.597,* p* < 0.001) (Fig. 3) defined cut-off thresholds for secondary fracture prediction at 16.5 kg and 24.5 kg, representing the lower and upper limits of grip strength. These thresholds categorized patients into high-risk, moderate-risk, and low-risk groups. Based on these thresholds, patients’ grip strength data were stratified into 3 strata: ≤ 16 kg (high-risk), 17–24 kg (moderate-risk), and ≥ 25 kg (low-risk) for secondary fracture risk (Table 3). Additionally, an age threshold of 74.5 years for the relative risk (RR) calculation was determined using receiver operating characteristic (ROC) curve analysis (AUC = 0.623,* p* < 0.001) (Appendix Fig. 6). This threshold was identified as the optimal cut-off point for distinguishing between high-risk and low-risk groups by age for secondary fractures following a distal radius fracture.

Patients aged over 74.5 years had a relative risk of secondary fracture over a mean 10-year follow-up of 1.64 (*p* < 0.001), while female sex carried a relative risk of 1.41 (*p* = 0.039) in the univariate analysis (Table 2). These factors showed significant associations with secondary fracture risk when considered individually. However, in the multivariate Cox proportional hazards regression analysis, with age and sex as controlled variables, neither age (OR = 1.01,* p* = 0.376) nor sex (OR = 0.77,* p* = 0.291) remained statistically significant. Instead, grip strength emerged as the independent predictor, with each 1 kg decrease linked to a 3.9% increase in secondary fracture risk (OR = 0.96, 95% CI = 0.93–0.99,* p* = 0.008) (Table 2).

**Table 2:** Univariate analysis and multivariate Cox proportional hazards regression analysis between age, sex, and grip strength, with age and sex as control variables in the multivariate analysis

	Univariate analysis: relative risk (RR) (95% CI)	***p***-value	Multivariate analysis: hazard ratio (HR) (95% CI)	***p***-value
Age (per year increase)	1.64 (95% CI = 0.99–1.02)	< 0.001	1.01 (95% CI = 0.99–1.02)	0.376
Sex (female vs. male)	1.41 (95% CI = 0.48–1.25)	0.039	0.77 (95% CI = 0.48–1.25)	0.291
Grip strength (per 1 kg increase)	-	-	0.96 (95% CI = 0.93–0.99)	0.008

While age and sex appear to be significant risk factors when considered individually, grip strength emerges as the most significant predictor when all factors are considered collectively in the multivariate analysis, with age and sex being controlled. A stronger grip strength was associated with a lower risk of re-fractures. Consequently, grip strength may serve as a more reliable indicator of secondary fracture risk, regardless of age or sex alone.

Patients with the weakest grip strength (≤ 16 kg) had the highest incidence and risk of subsequent fractures (*p* < 0.001) (Table 3). A significantly higher proportion (32.9%) of patients in the high-risk group (≤ 16 kg) had a history of re-fractures, compared to 19.9% of patients in the moderate-risk group (17–24 kg) and 14.8% of patients in the low-risk group (≥ 25 kg) (Table 3). The high-risk group (≤ 16 kg) had more than double the relative risk (RR) of secondary fractures compared to the low-risk group (≥ 25 kg), with an RR of 2.22 (95% CI = 1.55–3.17). The moderate-risk group (17–24 kg) had a 34% higher risk than the low-risk group (≥ 25 kg), with an RR of 1.34 (95% CI = 0.94–1.91) (Table 3). This indicates that patients with the lowest grip strength were more than twice as likely to experience a secondary fracture compared to those with higher grip strength (Table 3).

**Fig. 4:**
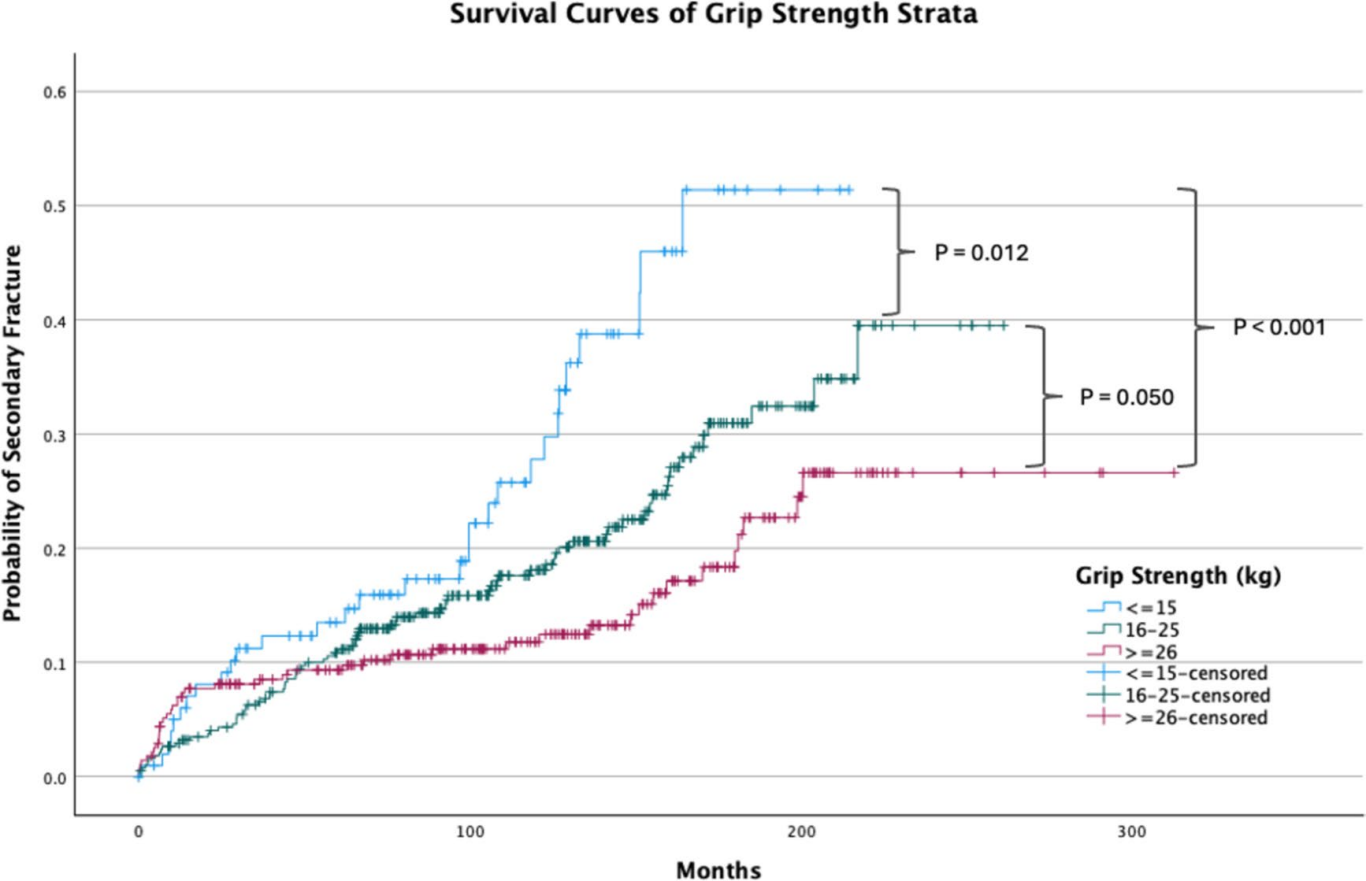
Survival/ time-to-event curves of grip strength strata

**Table 3:** Secondary fracture prediction by grip strength strata

	Risk	Risk Ratio (***RR***)	Secondary Fracture (***n***)			Mean Duration for Secondary Fracture (months)
Grip Strength			Yes	No	Total	
≤ 16 kg	High	2.22 (95% CI = 1.55 - 3.17)	48 (32.9%)	98 (67.1%)	146	168 (SE = 9.2)
17–24 kg	Moderate	1.34 (95% CI = 0.94 – 1.91)	60 (19.9%)	242 (80.1%)	302	208 (SE = 6.3)
≥ 25 kg	Low	1.00	44 (14.8%)	253 (85.2%)	297	260 (SE = 7.3)
Total			152 (20.4%)	593 (79.6%)	745	

Patients with the lowest grip strength (≤ 16 kg) also experienced the earliest occurrence of secondary fractures (*p* < 0.001) (Table 3). The Kaplan–Meier survival analysis further revealed a significant difference (*p* < 0.001) in the estimated mean duration to re-fracture across grip strength categories. Patients with grip strength ≤ 16 kg had an estimated mean duration of 168 months to re-fracture, while those with grip strength between 17 and 24 kg and ≥ 25 kg had longer estimated mean durations of 208 months and 260 months, respectively (Table 3).

Patients with the lowest grip strength (≤ 16 kg) showed the highest probability of secondary fractures at every time point post-DRF, except the first year (Fig. 4, Table 4). The Kaplan–Meier survival analysis (*p* < 0.001), revealed that patients with grip strength ≤ 16 kg had an estimated secondary fracture probability of 15.8% at 5 years, 30.4% at 10 years, and 54.0% at 15 years. In contrast, patients with grip strength ≥ 25 kg had lower estimated probabilities of 8.6% at 5 years, 11.3% at 10 years, and 19.6% at 15 years (Table 4). Pairwise comparison from the log-rank test showed statistical significance across all three grip strength strata (Fig. 4).

**Table 4:** Probability and number of cumulative cases of secondary fractures post-DRF according to grip strength strata

Grip strength	Risk	Probability of secondary fracture														
		Months														
		12	24	36	48	60	72	84	96	108	120	132	144	156	168	180
≤ 16 kg	High (*n* = 146)	0.048	0.077	0.120	0.142	0.158	0.191	0.210	0.220	0.278	0.304	0.373	0.389	0.454	0.507	0.540
17–24 kg	Moderate (*n* = 302)	0.030	0.040	0.057	0.089	0.107	0.117	0.130	0.144	0.149	0.166	0.191	0.207	0.233	0.265	0.278
≥ 25 kg	Low (*n* = 297)	0.064	0.075	0.075	0.086	0.086	0.098	0.102	0.107	0.107	0.113	0.119	0.126	0.152	0.172	0.196
Grip strength	Risk	Number of cumulative cases of secondary fracture (*n*)														
		Months														
		12	24	36	48	60	72	84	96	108	120	132	144	156	168	180
≤ 16 kg	High (*n* = 146)	7	11	17	20	21	26	28	29	34	36	41	42	45	47	48
17–24 kg	Moderate (*n* = 302)	9	12	17	26	31	34	37	40	41	44	48	50	53	56	57
≥ 25 kg	Low (*n* = 297)	19	22	22	25	25	28	29	30	30	31	32	33	36	38	40

A subgroup of 260 patients (63.5% females) had a history of DRF with hand muscle CT scans recorded in the PACS database (Fig. 1). Thenar muscle cross-sectional areas were available from 232 patients, of whom 33 (13.7%) had a history of secondary fractures. Hypothenar muscle cross-sectional areas were available for 234 patients, with 33 (14.1%) having a history of secondary fractures. The reliability of the muscle measurements for both the thenar and hypothenar muscles was high according to the intraclass correlation coefficient (ICC) (thenarICC = 0.963,* p* < 0.001; hypothenarICC = 0.934,* p* < 0.001).

**Fig. 5:**
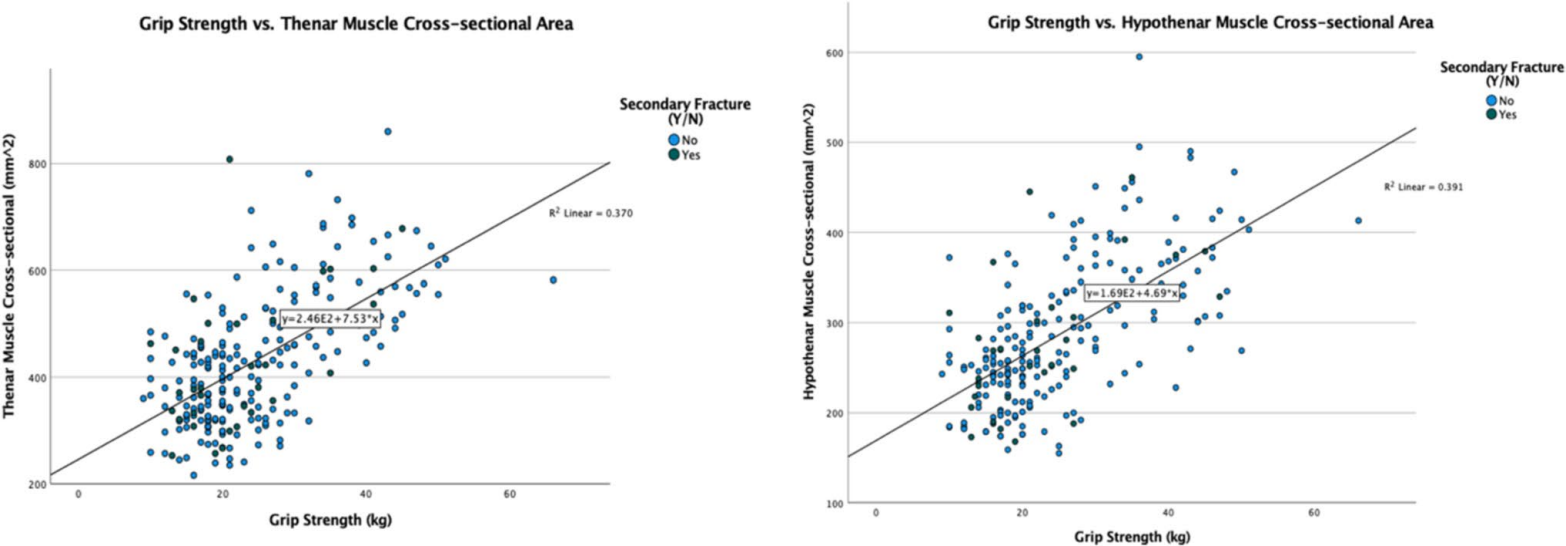
Scatterplot of grip strength and thenar/ hypothenar muscle cross-sectional area

Positive correlations were observed between grip strength and thenar/hypothenar cross-sectional areas (Fig. 5). (thenarr = 0.608,* p* < 0.001; hypothenar* r* = 0.625,* p* < 0.001).

Stratification of the thenar and hypothenar cross-sectional area for secondary fracture was attempted by ROC analysis, using the same approach as the grip strength analysis. However, statistical significance was not obtained (thenar* p* = 0.621; hypothenar* p* = 0.481) (Appendix Fig. 8). Consequently, further analyses on cross-sectional areas were not performed.

## Discussion

Our study demonstrated a strong association between low grip strength and increased risk of secondary fractures in patients with a history of distal radius fracture (DRF). While this relationship has been previously explored in the general population [30–32], there has been limited analysis specifically in DRF patients [1]. Our findings suggest that grip strength measurement serves as a useful surrogate for sarcopenia diagnosis and secondary fracture prediction in this population.

We found that patients in the high-risk group (≤ 16 kg grip strength) carry a 2.2-fold higher risk of secondary fractures compared to those in the low-risk group (≥ 25 kg). This aligns with a meta-analysis of 33 studies involving 45,926 individuals, which showed that sarcopenic patients have a 1.71-fold increase in clinical fractures than non-sarcopenic patients [38]. Our multivariate analysis identified grip strength as an independent predictor of secondary fractures with a statistically significant odd ratio of 0.961. This translates to a 4.1% higher likelihood of experiencing a secondary fracture per kg reduction in their grip strength, regardless of sex or age.

These findings are consistent with a prospective cohort study of a community-dwelling population in the same country, which found a 1.6-fold increase in clinical fractures with each standard deviation reduction in grip strength [32]. Similarly, another retrospective cohort study found that sarcopenia is a valid predictor of incident fractures for over an average follow-up of 11.3 years [12], which is comparable to our time-to-event analysis period of 5–15 years.

Kaplan–Meier survival analysis further corroborated these findings, showing statistically significant differences across all three grip strength strata (*p* < 0.001). At the 15-year mark, the probability of secondary fractures was substantially higher in the high-risk group (54.0%) compared to the low-risk group (19.6%). This marked difference underscores grip strength’s predictive value in long-term fracture risk assessment.

The 5-year probability of secondary fracture is 16% in our study comparable to the 19% reported by Dewan et al. in a prospective cohort study [39]. However, Hodsman et al. reported a lower 10-year probability of secondary fracture (14.2%) in a cohort of osteoporotic patients [6], suggesting potential variations between populations and highlighting the need for population-specific risk assessment.

The relationship between grip strength and fracture risk likely stems from the connection between muscle and bone health, often referred to as the muscle-bone unit [17]. This suggests that grip strength may serve as a proxy for overall musculoskeletal health, explaining its predictive value for fracture risk. The dynamic nature of grip strength testing may capture aspects of neuromuscular function and frailty not reflected in static measurements [27].

While bone mineral density (BMD) is useful for osteoporosis and sarcopenia [12], such investigation can significantly increase healthcare costs when performed on a large scale. Our study demonstrates that grip strength measurement offers a simple, cost-effective alternative for identifying patients at high risk of secondary fractures, potentially reducing the need for extensive BMD testing in all patients.

The implications for clinical practice are significant. Incorporating grip strength assessments into routine post-DRF care could provide a simple, cost-effective method for identifying patients at high risk of secondary fractures [27]. This could guide more targeted use of expensive imaging techniques and inform personalized prevention strategies, including nutrition [14], exercise programs [13], or pharmacological treatments [9] aimed at improving both muscle and bone health.

Interestingly, while grip strength showed a strong correlation with both thenar and hypothenar muscle cross-sectional areas (*r* = 0.608 and* r* = 0.625, respectively,* p* < 0.001 for both), these anatomical measurements did not prove to be significant predictors of secondary fractures when analyzed independently (thenar *p* = 0.621; hypothenar *p* = 0.481). These results suggest that grip strength is a more robust and clinically relevant predictor of secondary fracture risk compared to muscle cross-sectional area measurements. The strong correlation between grip strength and muscle area, coupled with the ease of measuring grip strength in clinical settings, further supports its use as a practical tool for risk stratification in post-DRF patients.

Our study has several limitations that should be considered. First, the absence of BMI and height data prevented us from normalizing the grip strength data and hand muscle cross-sectional areas, which could have provided more precise comparisons across patients. Second, the retrospective nature of the study may have introduced potential biases in data collection and analysis. Third, the lack of DEXA scans and bone mineral density (BMD) data limits our ability to directly compare grip strength with established measures of bone density. Additionally, the CT cross-sectional areas of hand muscles (thenar and hypothenar muscles) from patients with a history of DRF proved to be unreliable risk predictors for secondary fracture. This could be due to limitations such as limited sample size, inconsistent CT hand positioning, and large variation between patients possibly due to soft tissue swelling at the injured wrist [3].

Furthermore, we acknowledge the limitation of the uneven distribution of secondary fractures between genders (39 males vs. 112 females). This uneven distribution might have affected the results, as we did not stratify patients by gender in our analysis. Future research should consider this limitation and involve larger sample sizes to better understand the potential influence of gender on secondary fracture risk.

These limitations present opportunities for future research to further explore the relationship between osteosarcopenia and grip strength in DRF patients to address the current gaps in our understanding. For instance, incorporating comprehensive anthropometric measurements, bone density data, and long-term follow-up could further elucidate the relationship between grip strength, osteosarcopenia, and fracture risk in DRF patients [16]. Investigation into the effectiveness of grip strength-guided interventions in reducing secondary fracture risk could provide valuable insights for clinical [27]. Furthermore, studies comparing the predictive value of grip strength to other established risk factors for secondary fractures in DRF patients would help to position grip strength measurement within the broader context of fracture risk assessment tools [19].

## Conclusion

Our study demonstrates that grip strength is an effective predictor of secondary fracture risk in patients with a history of distal radius fracture (DRF). Our findings reveal that grip strength serves as a simple, cost-effective tool for risk stratification, independent of age and sex. We recommend integrating grip strength assessments into post-DRF care protocols to enhance risk evaluation and guide targeted preventive strategies.

Our research highlights the crucial link between muscle strength and bone health, emphasizing the concept of osteosarcopenia. While our study has limitations, it provides a foundation for future research to further validate grip strength as a predictor of secondary fractures.

Incorporating grip strength assessment into clinical practice could lead to more personalized and effective fracture prevention strategies for DRF patients, potentially reducing associated morbidity and healthcare costs.

## Figures and Tables

**Fig. 6:**
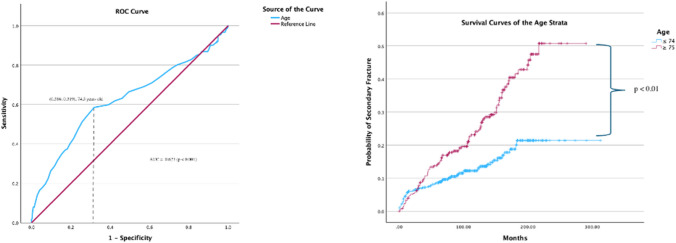
Receiver operating characteristic (ROC) analysis on the age data and corresponding survival / time-to-event curves

**Fig. 7:**
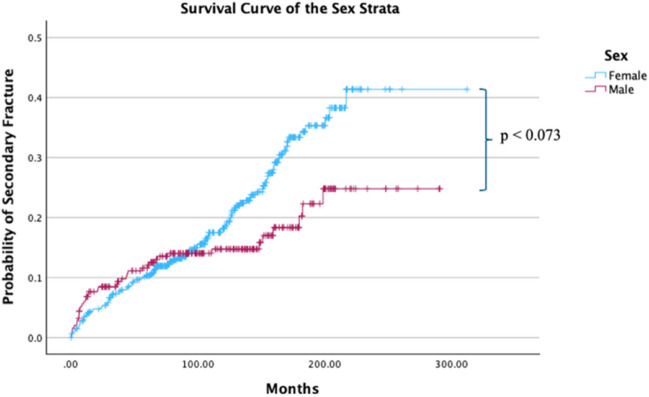
Survival / time-to-event curves of the sex strata female vs male

**Fig. 8:**
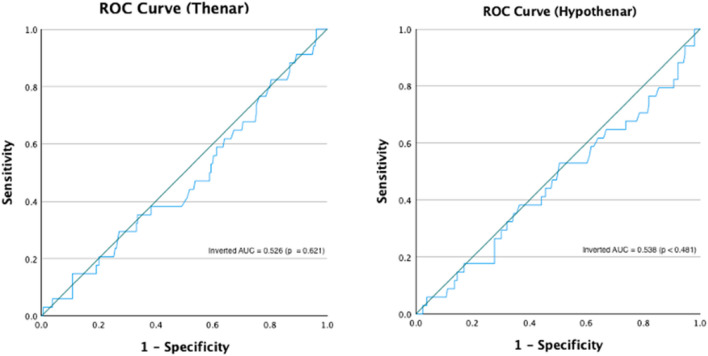
Receiver operating characteristic (ROC) analysis on thenar and hypothenar muscles

## Appendix

Table 5

**Table 5:** A summary of the types of secondary fractures included in the study

Types of 2nd fracture	Quantity (*n*)
Fracture of facial bones (802)	4
Fracture of vertebral column without mention of spinal cord injury (805)	12
Fracture of rib(s), sternum, larynx, and trachea (807)	7
Fracture of clavicle (810)	7
Fracture of scapula (811)	2
Fracture of humerus (812)	11
Fracture of radius and ulna (813)	41
Fracture of metacarpal bone(s) (815)	1
Fracture of one or more phalanges of the hand (816)	2
Fracture of neck of femur (820)	36
Fracture of other and unspecified parts of the femur (821)	6
Fracture of patella (822)	11
Fracture of tibia and fibula (823)	7
Fracture of ankle (824)	14
Fracture of one or more phalanges of the foot (826)	2
